# Corilagin Inhibits Neutrophil Extracellular Trap Formation and Protects against Hydrochloric Acid/Lipopolysaccharide-Induced Acute Lung Injury in Mice by Suppressing the STAT3 and NOX2 Signaling Pathways

**DOI:** 10.3390/antiox13040491

**Published:** 2024-04-19

**Authors:** Fu-Chao Liu, Huang-Ping Yu, Chia-Chih Liao, An-Hsun Chou, Hung-Chen Lee

**Affiliations:** 1 Department of Anesthesiology, Chang Gung Memorial Hospital, Linkou Branch, Taoyuan 333, Taiwan; ana5189@cgmh.org.tw (F.-C.L.); yuhp2001@cgmh.org.tw (H.-P.Y.); m7147@cgmh.org.tw (C.-C.L.); f5455@cgmh.org.tw (A.-H.C.); 2 College of Medicine, Chang Gung University, Taoyuan 333, Taiwan

**Keywords:** corilagin, neutrophil, neutrophil elastase, neutrophil extracellular trap, acute lung injury, STAT3, NOX2

## Abstract

Acute lung injury (ALI) and its severe manifestation, acute respiratory distress syndrome (ARDS), are characterized by uncontrolled inflammatory responses, neutrophil activation and infiltration, damage to the alveolar capillary membrane, and diffuse alveolar injury. Neutrophil extracellular traps (NETs), formed by activated neutrophils, contribute significantly to various inflammatory disorders and can lead to tissue damage and organ dysfunction. Corilagin, a compound found in Phyllanthus urinaria, possesses antioxidative and anti-inflammatory properties. In this study, we investigated the protective effects and underlying mechanisms of corilagin in hydrochloric acid (HCl)/lipopolysaccharide (LPS)-induced lung injury. Mice received intraperitoneal administration of corilagin (2.5, 5, or 10 mg/kg) or an equal volume of saline 30 min after intratracheal HCl/LPS administration. After 20 h, lung tissues were collected for analysis. Corilagin treatment significantly mitigated lung injury, as evidenced by reduced inflammatory cell infiltration, decreased production of proinflammatory cytokines, and alleviated oxidative stress. Furthermore, corilagin treatment suppressed neutrophil elastase expression, reduced NET formation, and inhibited the expression of ERK, p38, AKT, STAT3, and NOX2. Our findings suggest that corilagin inhibits NET formation and protects against HCl/LPS-induced ALI in mice by modulating the STAT3 and NOX2 signaling pathways.

## 1. Introduction

Acute lung injury (ALI) presents as a clinical syndrome characterized by inflammation and heightened pulmonary capillary permeability, potentially leading to acute respiratory distress syndrome (ARDS) in severe cases [1]. The characteristic hallmark of ALI is diffuse alveolar injury, marked by an uncontrolled inflammatory response, activation and infiltration of neutrophils, excessive production of proinflammatory cytokines, widespread apoptosis of lung epithelial cells, disruption of alveolar integrity, damage to the alveolar-capillary membrane, and compromised barrier functions [2]. Additionally, an injured capillary endothelium attracts neutrophils, which traverse the interstitial cavity to access the alveolar space filled with protein-rich edema fluid [3]. Despite therapeutic advances, documented mortality rates for ARDS range from 34 to 46%, contingent upon severity. ALI and ARDS persist as severe forms of diffuse lung disease, imposing a significant global health burden [4].

The etiology of ALI is diverse, encompassing factors such as severe infection, sepsis, trauma, shock, blood transfusion, and aspiration or inhalation of harmful substances [2]. Several experimental animal models have been established to investigate the mechanisms underlying direct or indirect ALI. In direct lung injury models, injury can be induced through intratracheal administration of bacteria or bacterial products such as lipopolysaccharide (LPS), acid administration such as hydrochloric acid (HCl), or the introduction of gastric particulates to simulate aspiration [5]. LPS, a key component in gram-negative bacterial outer membranes, strongly activates innate immune responses, resulting in lung epithelial cell apoptosis, rapid polymorphonuclear leukocyte (PMN) influx, and the release of inflammatory cytokines, reactive oxygen species (ROS), and chemotactic factors [6]. HCl aspiration, another PMN-dependent lung injury model, causes damage to the airway and alveolar epithelium, intra-alveolar and interstitial edema, alveolar hemorrhage, and compromised alveolar fluid clearance, closely resembling pathophysiological events in patients with pulmonary aspiration of gastric contents [7].

Neutrophils are essential components of the innate immune system, playing a central role in defending the host against microbial pathogen infections [8]. Upon activation, neutrophils produce excess superoxide anion (O_2_^•−^) and ROS, initiating acute inflammatory responses as they infiltrate tissues and organs. Concurrently, activated neutrophils form neutrophil extracellular traps (NETs) composed of neutrophil DNA and cytoplasmic proteins [9]. NETs have been demonstrated to be involved in various infectious, inflammatory, and autoimmune diseases [10]. Previous research highlights the critical role of NETs in injuries such as traumatic injury, ischemia–reperfusion-induced injury, and sepsis [11,12]. Prolonged and uncontrolled generation of ROS and NET formation emerge as crucial pathogenic factors in several inflammatory disorders. Studies suggest that NETs contribute to tissue damage and organ dysfunction by releasing proteases and neutrophil constituents extracellularly, thereby exacerbating injury [13]. This sequence results in NET formation, followed by cell death, a process collectively referred to as NETosis. The extent of NETosis appears to be associated with the magnitude of tissue damage and organ dysfunction [14].

Corilagin, a major polyphenolic constituent of Phyllanthus urinaria, a traditional herbal medicine, exhibits various pharmacological activities, including antioxidative, anti-inflammatory, and antiapoptotic effects [15]. Previous research has indicated that corilagin provides protection against LPS-induced liver injury, radiation-induced brain injury, and bleomycin-induced lung injury by mitigating oxidative stress and apoptosis pathways [16,17,18]. Recently, our study demonstrated its protective role in LPS-induced ALI, attributed to the attenuation of the NADPH oxidase 2 (NOX2) and extracellular signal-regulated kinase (ERK)/ nuclear factor kappa B (NF-κB) signaling pathways [19]. To date, there has been a lack of investigations into the pharmacological effects of corilagin in a combined HCl/LPS-induced ALI mouse model. Furthermore, the protective effects of corilagin on neutrophils and NETs expression in ALI and the underlying mechanisms have not been thoroughly explored. Thus, the present study aimed to elucidate these mechanisms.

## 2. Materials and Methods

### 2.1. Human Neutrophil Preparation

The purification protocol used in this study was approved by the Institutional Review Board of Chang Gung Memorial Hospital (IRB No. 202002189B0) and was conducted in accordance with the principles outlined in the Declaration of Helsinki (2013). Prior to blood collection, written informed consent was obtained from voluntary healthy donors aged 20–30 years. The separation of human neutrophils was performed following standardized procedures, which included dextran sedimentation followed by Ficoll-Hypaque density gradient centrifugation and lysis of erythrocytes.

### 2.2. Immunofluorescence Staining for Human Neutrophils

Human neutrophils were incubated at a concentration of 1 × 10^6^ cells/mL on glass slides precoated with fibrinogen (1 mg/mL) at 37 °C for 30 min. Subsequently, the neutrophils were treated with polyclonal antibodies against neutrophil elastase, histone H3, or Hoechst 33342 (1:100 dilution) and incubated overnight at 4 °C. Images of NETs were captured and analyzed using fluorescence microscopy.

### 2.3. Animals

Adult male C57BL/6C (B6) mice were procured from BioLASCO Taiwan Co., Ltd. (Taipei, Taiwan). The experimental protocols were approved by the Institutional Animal Care and Use Committee of Chang Gung Memorial Hospital (IACUC No. 2020121101).

### 2.4. Experimental Protocols

In the ALI model, mice were divided into six groups (*n* = 6/group): control (saline), corilagin alone (10 mg/kg), HCl/LPS (0.2 N HCl + 2.5 μg/g LPS in 50 μL PBS), HCl/LPS + corilagin (2.5 mg/kg), HCl/LPS + corilagin (5 mg/kg), and HCl/LPS + corilagin (10 mg/kg). Corilagin or an equal volume of saline was administered intraperitoneally 30 min after intratracheal HCl/LPS administration. Mice were euthanized 20 h after HCl/LPS challenge, and lung tissues were collected for subsequent analyses.

### 2.5. Histology and Immunohistochemistry

The lungs were excised, fixed in 4% paraformaldehyde, and embedded in paraffin. Histological examination was performed on 4 μm thick sections stained with hematoxylin and eosin. Immunohistochemical staining was conducted by incubating sections with primary antibodies against Ly-6G (neutrophil; 1:500), Mac-2 (macrophage; 1:500; eBioscience, Inc., San Diego, CA, USA), or neutrophil elastase (1:500; Abcam, Cambridge, MA, USA).

### 2.6. Expression of Neutrophil Extracellular Traps (NETs)

For neutrophil elastase staining, a goat anti-rabbit IgG H&L (FITC) secondary antibody was applied for 1 h at room temperature (RT) (1:1000). Histone H3 was stained with a goat anti-mouse IgG H&L (TRITC) secondary antibody for 1 h at RT (1:1000). Following staining, the samples were treated with 1 ng/mL Hoechst 33342 for 10 min at RT and subsequently washed in PBS three times. Confocal laser scanning fluorescence microscopy (LSM 510 META; Carl Zeiss, Oberkochen, Germany) was used for analysis.

### 2.7. Measurement of TNF-α and IL-6 Levels in Lung Tissue

Lung tissues were homogenized, and the resulting supernatants were utilized to quantify tumor necrosis factor alpha (TNF-α) and interleukin (IL)-6 concentrations using ELISA kits (R&D Systems, Minneapolis, MN, USA) and a spectrometer (absorbance at 450 nm). Each ELISA sample was analyzed in duplicate, and the cytokine levels were normalized based on tissue weight.

### 2.8. Measurement of Tissue MDA and GSH Levels

Lung tissues were homogenized in cold Tris-HCl buffer on ice, followed by centrifugation at 10,000× *g* for 15 min at 4 °C. The resulting deproteinized supernatants were utilized to quantify malondialdehyde (MDA) levels using a Bioxytech MDA-586 assay kit (OxisResearch, Portland, OR, USA) and to determine glutathione (GSH) levels using a glutathione assay kit (Cayman Chemical, Ann Arbor, MI, USA), following the manufacturers’ instructions.

### 2.9. Western Blotting

The tissue preparation procedure followed previously described methods. The membranes were incubated overnight at 4 °C with antibodies against ERK, phospho-ERK, p38, phospho-p38, protein kinase B (AKT), phospho-AKT, signal transducer and activator of transcription 3 (STAT3), phospho-STAT3, and NOX2 (Cell Signaling Technology Inc., Beverly, MA, USA) at a dilution of 1:1000.

### 2.10. Statistical Analysis

The data are expressed as the mean ± standard error of the mean (SEM) for each group, with 6 mice per group. Statistical analysis was conducted using one-way analysis of variance (ANOVA) followed by Tukey’s multiple comparison tests. Prism 6.0 Software (GraphPad Software Inc., San Diego, CA, USA) was used for the statistical analyses. A *p*-value < 0.05 was considered to indicate statistical significance.

## 3. Results

### 3.1. Effects of Corilagin on the Formation of NETs in Activated Human Neutrophils

**Figure 1:**
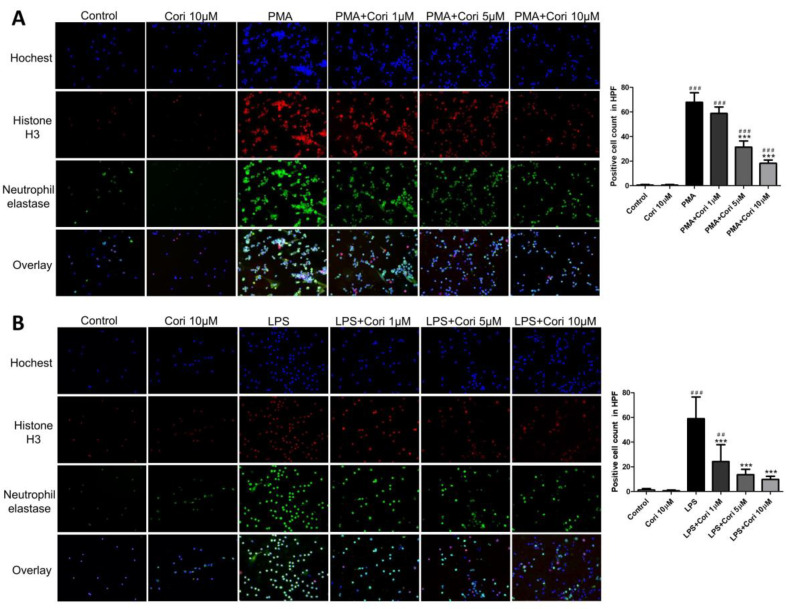
Effects of corilagin on formation of NETs in activated human neutrophils induced by PMA (**A**) or LPS (**B**). Human neutrophils were incubated with corilagin (1, 5, and 10 uM), or equal volume of saline was administered for 10 min and then activated by PMA (10 nM) or LPS (100 μg/mL). Representative overlay expression is shown. Immuonofluorescence imaging shows NETs labelled with neutrophil elastase (green) and histone 3 (red). DNA were stained with hochest 33342 (blue) (magnification, ×200). Quantification of positive cells was analyzed under high power field (HPF). All data are represented as mean ± SEM; *n* = 6 for each group. ## *p* < 0.01, ### *p* < 0.005 vs. control; *** *p* < 0.005 vs. PMA or LPS.

To assess the impact of corilagin on NET formation in activated human neutrophils, we exposed human neutrophils to corilagin (1, 5, and 10 μM) or an equivalent volume of saline for 10 min, followed by activation with phorbol myristate acetate (PMA, 10 nM) or LPS (100 μg/mL). The PMA or LPS-treated groups exhibited substantial NET formation compared to the control group. Conversely, the corilagin-treated groups at concentrations of 1, 5, and 10 μM showed a dose-dependent reduction in NET formation compared to the PMA or LPS group (Figure 1). These findings indicate that corilagin treatment mitigated NET formation in activated human neutrophils induced by PMA or LPS.

### 3.2. Effects of Corilagin on Histological Changes in the Lung following HCl/LPS-Induced Lung Injury

**Figure 2:**
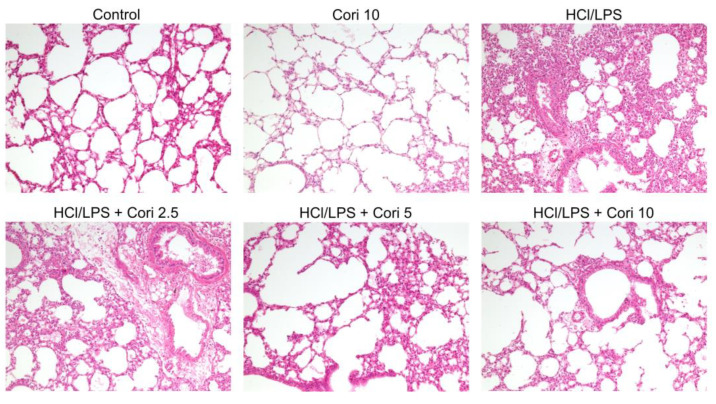
Effects of corilagin on histological changes in HCl/LPS-induced lung injury. Corilagin (Cori, 2.5, 5, and 10 mg/kg), or an equivalent volume of saline, was intraperitoneally administered 30 min after intratracheal challenge with HCl/LPS. Mice were euthanized after 20 h of HCl/LPS exposure, and lung tissues were collected. Representative histological images of hematoxylin and eosin staining (magnification, ×100).

To assess the impact of corilagin on ALI, lung tissues were collected from the mice 20 h after intratracheal HCl/LPS challenge and subsequently fixed and stained with H&E. The HCl/LPS-exposed group exhibited notable histopathological alterations, including alveolar wall thickening, interstitial edema, and pulmonary congestion, in contrast to the control group. However, treatment with corilagin (2.5, 5, and 10 mg/kg) administered 30 min after HCl/LPS challenge mitigated these histopathological changes (Figure 2). These findings suggest that corilagin has the potential to alleviate HCl/LPS-induced lung injury.

### 3.3. Effects of Corilagin on the Infiltration of Neutrophils and Macrophages in HCl/LPS-Induced Lung Injury

**Figure 3:**
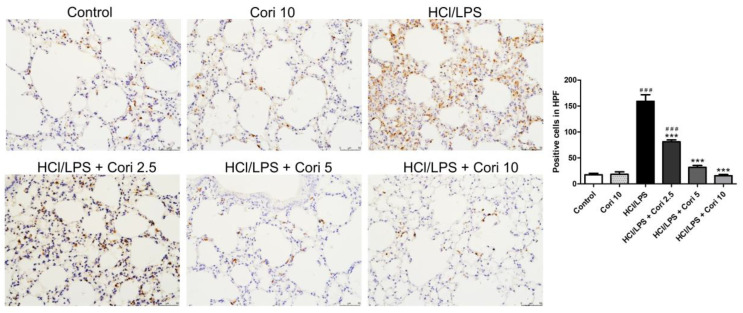
Effects of corilagin on neutrophil infiltration in HCl/LPS-induced lung injury. Corilagin (Cori, 2.5, 5, and 10 mg/kg), or an equivalent volume of saline, was intraperitoneally administered 30 min after intratracheal challenge with HCl/LPS. Mice were euthanized after 20 h of HCl/LPS exposure, and lung tissues were collected. Representative images of immunohistochemical staining for Ly6G (magnification, ×200). Quantification of positive cells was analyzed HPF. All data are represented as mean ± SEM; *n* = 6 for each group. ### *p* < 0.005 vs. control group; *** *p* < 0.005 vs. the HCl/LPS group.

**Figure 4:**
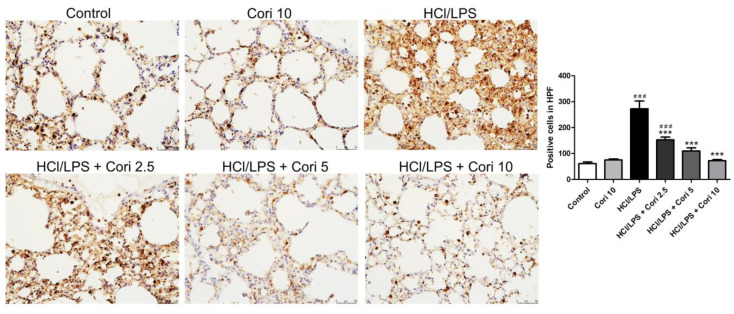
Effects of corilagin on macrophage infiltration in HCl/LPS-induced lung injury. Corilagin (Cori, 2.5, 5, and 10 mg/kg), or an equivalent volume of saline, was intraperitoneally administered 30 min after intratracheal challenge with HCl/LPS. Mice were euthanized after 20 h of HCl/LPS exposure, and lung tissues were collected. Representative images of immunohistochemical staining for Mac-2 (magnification, ×200). Quantification of positive cells was analyzed under HPF. All data are represented as mean ± SEM; *n* = 6 for each group. ### *p* < 0.005 vs. control group; *** *p* < 0.005 vs. the HCl/LPS group.

To assess neutrophil infiltration in HCl/LPS-induced lung injury, lung sections were immunohistochemically stained with Ly6G, a granulocyte-specific marker. The HCl/LPS-exposed group exhibited prominent neutrophil infiltration in the lung tissue compared to the control group. Conversely, the corilagin-treated groups (2.5, 5, and 10 mg/kg) exhibited markedly reduced neutrophil accumulation in lung tissue compared to the HCl/LPS group (Figure 3). Similarly, to examine macrophage infiltration in ALI, lung sections were immunohistochemically stained with Mac-2, a macrophage-specific marker. The HCl/LPS-exposed group displayed evident macrophage infiltration in the lung tissue compared to the control group. Conversely, the corilagin-treated groups (2.5, 5, and 10 mg/kg) exhibited significantly reduced macrophage accumulation in lung tissue compared to the HCl/LPS group (Figure 4).

### 3.4. Effects of Corilagin on Pneumonic TNF-α and IL-6 Levels in HCl/LPS-Induced Lung Injury

**Figure 5:**
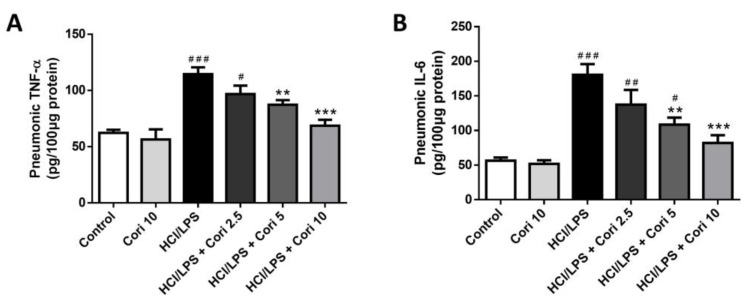
Effects of corilagin on pneumonic TNF-α (**A**) and IL-6 (**B**) levels in HCl/LPS-induced lung injury. Corilagin (Cori, 2.5, 5, and 10 mg/kg), or an equivalent volume of saline, was intraperitoneally administered 30 min after intratracheal challenge with HCl/LPS. Mice were euthanized after 20 h of HCl/LPS exposure, and lung tissues were collected for ELISA. All data are represented as mean ± SEM (*n* = 6 per group). # *p* < 0.05, ## *p* < 0.01, ### *p* < 0.005, vs. the control group; ** *p* < 0.01, *** *p* < 0.05 vs. the HCl/LPS group.

To assess the impact of corilagin on proinflammatory cytokine expression in HCl/LPS-induced lung injury, we quantified TNF-α and IL-6 levels in lung tissues using ELISA. The pulmonary concentrations of TNF-α and IL-6 were significantly greater in the HCl/LPS group than in the control group. No significant differences in cytokine levels were observed between the control group and corilagin-alone group. However, treatment with higher doses of corilagin (5 and 10 mg/kg) administered 30 min after HCl/LPS challenge notably attenuated pulmonary TNF-α (*p* < 0.01, *p* < 0.005, respectively) and IL-6 (*p* < 0.01, *p* < 0.005, respectively) levels compared to the HCl/LPS group (Figure 5). These findings suggest that corilagin treatment mitigates the production and expression of proinflammatory cytokines in HCl/LPS-induced lung injury.

### 3.5. Effects of Corilagin on Pneumonic MDA and GSH Levels in HCl/LPS-Induced Lung Injury

**Figure 6:**
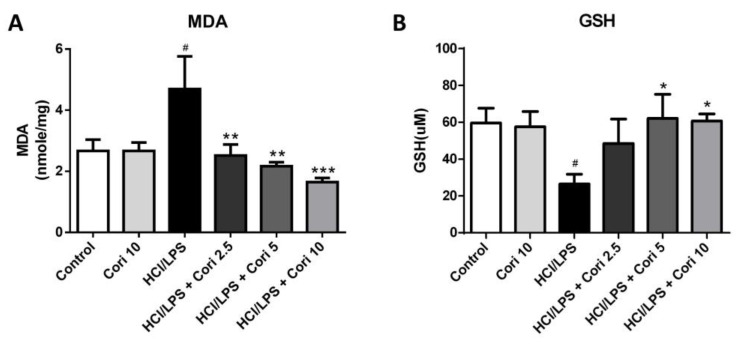
Effects of corilagin on pneumonic malondialdehyde (**A**) and glutathione (**B**) levels in HCl/LPS-induced lung injury. Corilagin (Cori, 2.5, 5, and 10 mg/kg), or an equivalent volume of saline, was intraperitoneally administered 30 min after intratracheal challenge with HCl/LPS. Mice were euthanized after 20 h of HCl/LPS exposure, and lung tissues were collected. All data are represented as mean ± SEM (*n* = 6 per group). # *p* < 0.05 vs. the control group; * *p* < 0.05, ** *p* < 0.01, *** *p* < 0.05 vs. the HCl/LPS group.

We assessed alterations in oxidative stress markers. As depicted in Figure 6, the levels of MDA, a marker of lipid peroxidation, were notably elevated in the HCl/LPS group compared to the control group (*p* < 0.05). However, treatment with corilagin (2.5, 5, and 10 mg/kg) significantly reduced these levels compared to the HCl/LPS group (*p* < 0.01, *p* < 0.01, and *p* < 0.005, respectively). Additionally, the levels of GSH, a cellular antioxidant marker, were substantially lower in the HCl/LPS group compared to the control group (*p* < 0.05). Yet, treatment with 5 and 10 mg/kg corilagin significantly elevated GSH levels compared to the HCl/LPS group (*p* < 0.05 and *p* < 0.05, respectively). These findings underscore the antioxidative effects of corilagin on HCl/LPS-induced lung injury.

### 3.6. Effects of Corilagin on Pneumonic Neutrophil Elastase Expression in HCl/LPS-Induced Lung Injury

**Figure 7:**
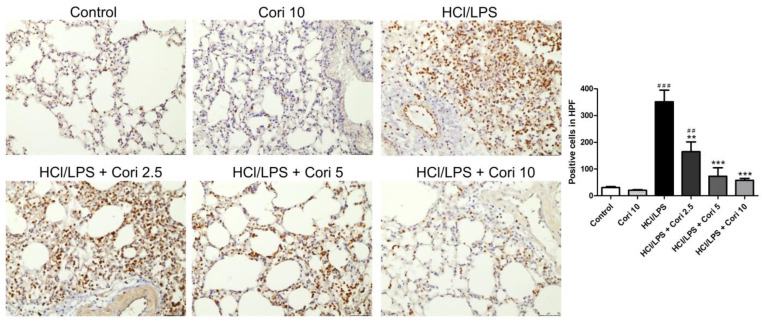
Effects of corilagin on pneumonic neutrophil elastase expression in HCl/LPS-induced lung injury. Corilagin (Cori, 2.5, 5, and 10 mg/kg), or an equivalent volume of saline, was intraperitoneally administered 30 min after intratracheal challenge with HCl/LPS. Mice were euthanized after 20 h of HCl/LPS exposure, and lung tissues were collected. Representative images of immunohistochemical staining for neutrophil elastase (magnification, ×200). Quantification of positive cells was analyzed under HPF. All data are represented as mean ± SEM; *n* = 6 for each group. ## *p* < 0.01, ### *p* < 0.005 vs. control group; ** *p* < 0.01, *** *p* < 0.005 vs. the HCl/LPS group.

To explore the potential anti-inflammatory mechanism of corilagin in HCl/LPS-induced lung injury, lung sections were immunohistochemically stained with neutrophil elastase antibody. In comparison to the control group, the HCl/LPS group exhibited notable neutrophil elastase expressions in lung tissues. Conversely, the corilagin-treated groups (2.5, 5, and 10 mg/kg) displayed significantly reduced neutrophil elastase expressions in the lung tissues compared to the HCl/LPS group (Figure 7).

### 3.7. Effects of Corilagin on the Formation of NETs in HCl/LPS-Induced Lung Injury

**Figure 8:**
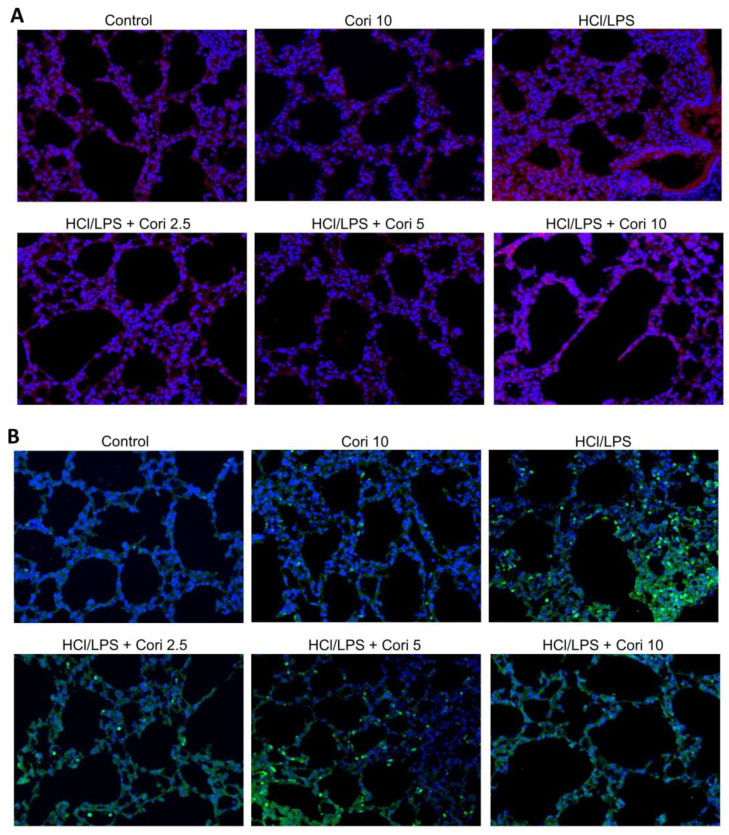
Effects of corilagin on formation of NETs in HCl/LPS-induced lung injury. Corilagin (Cori, 2.5, 5, and 10 mg/kg), or an equivalent volume of saline, was intraperitoneally administered 30 min after intratracheal challenge with HCl/LPS. Mice were euthanized after 20 h of HCl/LPS exposure, and lung tissues were collected. Histologic images of histone 3 (**A**) and neutrophil elastase (**B**) staining (200× objective lens field) groups. Panel shows the triple immunofluorescence staining of nucleic acid (Hoechst 33342, blue), neutrophil elastase (FITC-labeled, green) and histone H3 (TRITC-labeled, red) following a 20 h acute lung injury.

We further assessed the impact of corilagin on NET formation in HCl/LPS-induced lung injury using immunofluorescence staining. No significant differences in NET formation were observed between the control group and corilagin alone group. However, there was a significant increase in NET formation in the HCl/LPS group compared to the control group. Furthermore, the administration of corilagin (2.5, 5, and 10 mg/kg) 30 min after HCl/LPS challenge markedly reduced NET formation compared to the HCl/LPS group (Figure 8A,B). These findings collectively suggest that corilagin treatment attenuates NET formation in HCl/LPS-induced lung injury.

### 3.8. Effects of Corilagin on Pneumonic ERK, p38, AKT, STAT3, and NOX2 Expression in HCl/LPS-Induced Lung Injury

**Figure 9:**
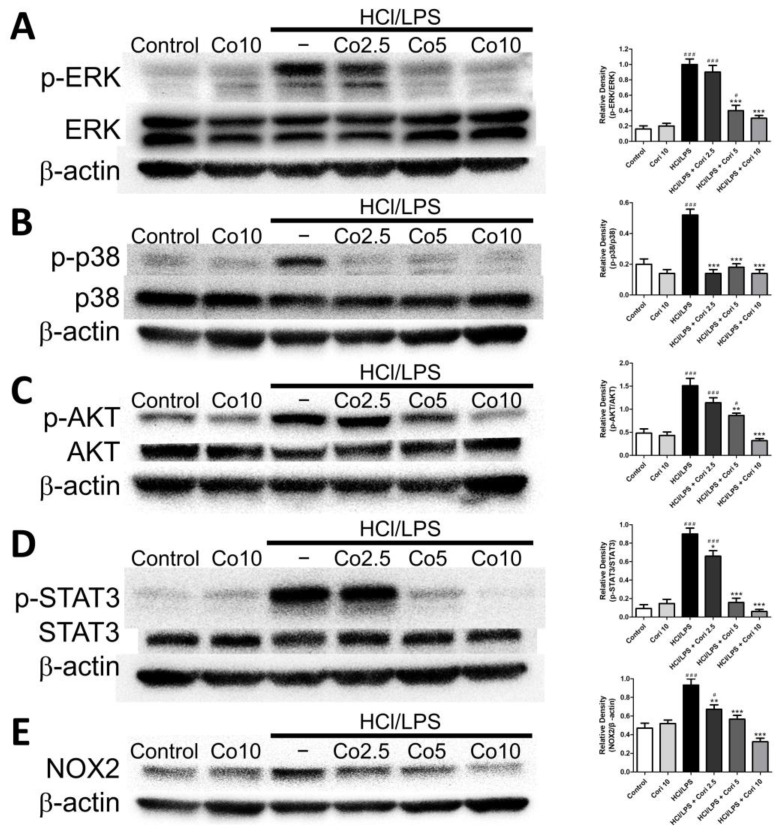
Effects of corilagin on pneumonic ERK (**A**), p38 (**B**), AKT (**C**), STAT3 (**D**), and NOX2 (**E**) expression in HCl/LPS-induced lung injury. Corilagin (Cori, 2.5, 5, and 10 mg/kg), or an equivalent volume of saline, was intraperitoneally administered 30 min after intratracheal challenge with HCl/LPS. Mice were euthanized after 20 h of HCl/LPS exposure, and lung tissues were collected for western blot. The bands were analyzed using densitometry. All data are represented as mean ± SEM. # *p* < 0.05, ### *p* < 0.005 vs. control group; * *p* < 0.05, ** *p* < 0.01, *** *p* < 0.005 vs. the HCl/LPS group.

We further investigated the expression of pneumonic ERK, p38, AKT, STAT3, and NOX2 in HCl/LPS-induced lung injury. There were no significant differences in the expression of pneumonic ERK, p38, AKT, STAT3, and NOX2 proteins between the control group and corilagin alone group. However, the levels of phosphorylated ERK, p38, AKT, STAT3, and NOX2, indicative of their activity, were elevated in the HCl/LPS group compared to the control group. Additionally, treatment with corilagin (20 and 40 mg/kg) administered 30 min after HCl/LPS challenge significantly reduced the expression of phosphorylated ERK, p38, AKT, STAT3, and NOX2 compared to the HCl/LPS group (Figure 9). These results suggest that corilagin treatment attenuates the activation of ERK, p38, AKT, STAT3, and NOX2 in HCl/LPS-induced lung injury.

## 4. Discussion

In this study, we initially explored the impact of corilagin on NET formation in activated human neutrophils. Subsequently, we systematically assessed the protective effects of corilagin against HCl/LPS-induced ALI in a murine model and elucidated the underlying mechanisms involved. Our findings indicated that corilagin treatment effectively mitigated NET formation in PMA- or LPS-activated human neutrophils. In the HCl/LPS-induced ALI mouse model, corilagin treatment notably attenuated lung injury, as evidenced by reduced inflammatory cell infiltration, diminished production of proinflammatory cytokines, and alleviated oxidative stress. Furthermore, corilagin treatment significantly suppressed neutrophil elastase expressions, decreased NET formation, and inhibited the expression of ERK, p38, AKT, STAT3, and NOX2. Collectively, our study highlights that corilagin exhibits inhibitory effects on NET formation and confers protection against HCl/LPS-induced ALI in mice, potentially through the modulation of the STAT3 and NOX signaling pathways.

The pathogenesis of ALI involves the compromise of alveolar–capillary membrane integrity, leading to increased permeability and the influx of protein-rich fluid into alveolar spaces [1]. Microvascular endothelial injury initiates this process, enabling neutrophils to adhere and migrate through the interstitium into the airspace [20]. Transepithelial neutrophil migration is pivotal, because these cells play a central role in inflammation [21]. Excessive or prolonged neutrophil activation contributes to basement membrane destruction and heightened permeability, releasing proinflammatory mediators [22]. Alveolar macrophages secrete cytokines, such as IL-1, IL-6, and TNF-α, locally stimulating chemotaxis and recruiting inflammatory cells [23]. Ultimately, the influx of protein-rich edema fluid disrupts surfactant function, leading to dysfunction and inactivation, culminating in ALI and, in severe cases, ARDS [24]. In the present study, corilagin treatment markedly mitigated pulmonary parenchymal injury, suppressed neutrophil and macrophage infiltration, and reduced the production of proinflammatory cytokines. Our findings align with the existing literature, reinforcing the notion that corilagin provides protection against HCl/LPS-induced ALI through its anti-inflammatory properties.

Various modeling strategies have been developed to replicate human ALI/ARDS in animal models [25]. Direct lung injury models involve intratracheal administration of LPS, acid aspiration, lung ischemia–reperfusion, and mechanical ventilation-associated injury. Indirect lung injury models aim to mimic sepsis, including intravenous LPS administration, cecal ligation and puncture (CLP), and mesenteric ischemia–reperfusion [5]. However, sepsis-induced lung injury may not faithfully replicate human ALI due to decreased intra-alveolar neutrophil infiltration, proteinaceous alveolar edema, and limited involvement of alveolar spaces [26]. In this study, we utilized direct lung injury models involving intratracheal LPS administration, which effectively induces neutrophil infiltration into the lungs, and HCl aspiration, known for inducing epithelial injury and barrier disruption [27]. The primary objective of our study was to investigate whether corilagin can protect against ALI by inhibiting neutrophil infiltration and NET formation. Consequently, we selected a combined HCl and LPS intratracheal administration model to induce lung injury. Histopathological analysis revealed characteristics such as intra-alveolar neutrophil infiltration, neutrophilic inflammation, interstitial edema, and necrosis.

Neutrophils, the foremost innate immune cells, swiftly migrate to sites of tissue inflammation, constituting a crucial component of the innate immune response [28]. Functioning as part of the frontline defense, neutrophils are the first immune cells to mobilize to sites of inflammation, playing pivotal roles in pathogen eradication and cytokine secretion [8]. Upon activation at the inflammatory site, neutrophils serve as signaling mediators and execute various antimicrobial functions, including phagocytosis, cytokine release, and degranulation. Recent studies have unveiled the formation of NETs as a novel defense mechanism. NETs, comprising net-like chromatin fibers filled with neutrophil-derived antimicrobial proteins, arise from dying neutrophils through a process known as NETosis [29]. They immobilize pathogens, impeding infection dissemination and facilitating pathogen elimination [30]. However, the excessive presence of NETs, particularly NET-bound components, can yield detrimental effects. Histones and myeloperoxidase (MPO) exhibit cytotoxicity to epithelial and endothelial cells, while neutrophil elastase (NE) disrupts alveolar–capillary barrier integrity by cleaving the endothelial actin cytoskeleton [31]. Moreover, mounting evidence suggests that NETosis is increased in both infectious and sterile ALI conditions [32]. Excessive NET release promotes thrombus formation, activates endothelial cells, and induces microvascular injury, resulting in heightened vascular permeability, intra-alveolar accumulation of protein-rich fluid, and inflammatory cell infiltration [33]. In our current investigation, we initially established that corilagin treatment effectively reduces NET formation in PMA- or LPS-activated human neutrophils. Additionally, we observed a significant increase in NET expression in the lungs of mice following HCl/LPS-induced lung injury. Remarkably, corilagin treatment substantially mitigated the expression of histone 3 and elastase in this model. Collectively, our findings strongly indicate that corilagin exerts protective effects in ALI, possibly through the inhibition of NET formation.

The mitogen-activated protein kinase (MAPK) protein family, crucial for various cellular processes including oxidative stress, survival, apoptosis, and inflammation, plays a pivotal role in ALI [34]. Specifically, the ERK, jun N-terminal kinase (JNK), and p38 pathways are implicated in NF-κB activation, prompting the production of proinflammatory cytokines and mediators that exacerbate lung inflammation in ALI [19]. Additionally, AKT signaling pathways, essential for cell survival, proliferation, apoptosis, and inflammatory responses, are involved in LPS-induced lung injury, modulating oxidative stress and inflammation [35]. Moreover, STAT3, activated by proinflammatory cytokines such as IL-1β and IL-6, regulates inflammation, survival, differentiation, and proliferation [36,37]. Recent studies have shown the protective effect of STAT3 inhibition against LPS-induced ALI through a reduction in inflammatory cell infiltration and proinflammatory gene expression [38,39,40,41]. Furthermore, recent research has highlighted the potential importance of the STAT3 axis in facilitating NET release in a sepsis model and its contribution to sepsis-associated encephalopathy [42]. Our previous studies demonstrated that corilagin possesses protective and anti-inflammatory effects on acetaminophen-induced liver injury through the IL-6/STAT3 signaling pathway [43]. In this study, our findings revealed elevated expression of ERK, p38, AKT, and STAT3 in HCl/LPS-induced ALI, and corilagin treatment effectively mitigated the phosphorylation and expression. These findings collectively suggest that corilagin exerts protective effects against ALI by suppressing the MAPKs, AKT, and STAT3 signaling pathways, in line with its modulation of TNF-α and IL-6 levels.

NOX2, an inaugural member of the NOX family, has undergone extensive biochemical and physiological scrutiny. It acts as a primary source of ROS in neutrophils, macrophages, and endothelial cells, playing a pivotal role in phagocytosis and inflammation-related injury by releasing ROS in various inflammatory conditions [44]. However, excessive NOX2 activation in the lungs has been associated with tissue injury, particularly in ALI, where it fosters NF-κB-dependent acute inflammatory responses, neutrophil infiltration, and lung tissue damage [45]. Recent investigations have revealed that NOX2 inhibitors significantly curtail ROS production and alleviate lung injury in LPS-induced ALI models [46]. Furthermore, our recent findings indicate that corilagin treatment attenuates acetaminophen-induced liver injury by inhibiting NOX-derived ROS [43]. Additionally, the activation of NOX2 and the NOX2-derived ROS-dependent pathway are pivotal for NET formation and oxidative defense against pathogens following neutrophil activation. ROS formation triggers the release of neutrophil elastase and MPO from neutrophil granules, facilitating their translocation into the cell nucleus and inducing chromatin decondensation, thereby releasing NETs [47,48]. In the present investigation, the significant increases in NOX2 levels and NET formations observed following HCl/LPS-induced lung injury were notably reduced by corilagin treatment. These findings collectively suggest that corilagin possesses antioxidant properties and inhibits NET formation, potentially through the downregulation of NOX2 expression in the lungs.

The present study has some limitations. Notably, we did not employ specific inhibitors targeting each pathway or utilize transgenic mice in our experimental design. Further investigations are warranted to elucidate whether corilagin directly or indirectly suppresses the STAT3 and NOX pathways in this HCl/LPS-induced ALI model.

## 5. Conclusions

In conclusion, corilagin has protective effects against HCl/LPS-induced ALI in mice by mitigating inflammatory immune cell infiltration, suppressing the production of proinflammatory cytokines, and mitigating oxidative stress. The anti-inflammatory and antioxidant effects of corilagin appear to be linked to its inhibitory effects on NET formation, possibly mediated through the STAT3 and NOX2 signaling pathways. These findings suggest that corilagin is a promising therapeutic intervention for ALI and ARDS, while NETs are potential therapeutic targets for addressing neutrophil-driven injury and inflammatory conditions.

## Data Availability

The data presented in this study are available on request from the corresponding author.
